# Cyclin-dependent kinase 5 acts as a promising biomarker in clear cell Renal Cell Carcinoma

**DOI:** 10.1186/s12885-019-5905-9

**Published:** 2019-07-16

**Authors:** Liangsong Zhu, Rong Ding, Jianping Zhang, Jin Zhang, Zongming Lin

**Affiliations:** 1 Department of Urology, Zhongshan Hospital, Fudan University, 180 Fenglin Road, Shanghai, China; 2 Department of Obstetrics and Gynecology, International Peace Maternity and Child Health Hospital, School of Medicine, Shanghai Jiao Tong University, Shanghai, China; 3 Department of Urology, Ren Ji Hospital, School of Medicine, Shanghai Jiaotong University, 1630 Dong Fang Road, Shanghai, China

**Keywords:** CDK5, p21, ccRCC, Prognostic

## Abstract

**Background:**

This research provides the first evidence of CDK5 in ccRCC prognosis and correlation with different p21 expression in overall survival (OS) analysis.

**Methods:**

The data from both of The Cancer Genome Atlas (TCGA) and Gene Expression of Normal and Tumor Tissue (GENT) were analyzed for determining the expression of CDK5 in kidney cancer. Tissue microarray that made by using 150 ccRCC samples was used in immunohistochemistry (IHC) analysis. A validation of OS cohort was extracted from Oncomine database.

**Results:**

The CDK5 expression was significantly lower in cancer tissue compared with normal in TCGA (*p* < 0.0001), GENT database also showed a relative low expression in kidney cancer. Among 150 ccRCC patients, low CDK5 was detected in 83 cases (55.3%), low p21 in 97 cases (64.7%). CDK5 was associated with the advanced TNM stage (*p* = 0.042), and Fuhrman grade (*p* = 0.035). Patients with lower CDK5 might be more likely to be aggressive status. According to the combination analysis of CDK5 and p21, patients in CDK5 low/p21 low group showed poorer survival rate, and no significant survival difference was observed in other groups. In the Cox multivariate analysis, the co-expression of CDK5 low/p21 low was identified as an independent prognostic factor in ccRCC patients.

**Conclusions:**

Together, our findings provided the first evidence that CDK5 was acting as a promising biomarker in ccRCC patients, and co-expression of CDK5 and p21 is an independent prognostic for overall survival. IHC analysis of CDK5 and p21 on cancer tissues after surgery may help to evaluate and predict the outcome of ccRCC patients.

**Electronic supplementary material:**

The online version of this article (10.1186/s12885-019-5905-9) contains supplementary material, which is available to authorized users.

## Background

The incidence of renal cell carcinoma (RCC) has been increased in the past decades, RCC is also reported to be the 14th most common malignancy and most lethal urologic cancer [1, 2]. Nowadays, some researches reported that several environmental risk factors have been identified for the development of RCC, including hypertension, smoking, obesity, and diabetes [3, 4]. The clear cell renal cell carcinoma (ccRCC) is the most common pathological subtype which nearly account for 70–75%, the papillary RCC account for 10–16%, and chromophone RCC account for 5%. Since the Von Hippel-Lindau (VHL) disease tumor suppressor gene *VHL* is commonly inactivated in ccRCC, the tyrosine kinase inhibitors (TKIs) that modulate the *pVHL*-*HIF*-*VEGF* signaling pathway have showed treating benefit in patients with advanced ccRCC. Currently, TKIs such as sunitinib and sorafenib have been approved as the standard treating strategy for metastatic ccRCC [5]. However, there is still a subgroup of patients who have no response to such therapy and a number of patients have resistance over time. Therefore, it is of great importance to explore new molecular markers that will help us to evaluate the treating response and prognosis, furthermore, to develop novel therapies.

Cyclin-dependent kinase 5 (CDK5), a serine/threonine kinase, is a member of CDKs, but it is unique among common CDKs with no cell cycle or mitotic function because of lacking classical mediator of cell-cycle transition [6]. Previous studies have identified that CDK5 is important in neuronal development, neuronal function, and neuronal disease [7]. The investigation of CDK5 function in extra-neuronal tissues is increasing as well [8], especially in cancer research. Recently, emerging evidence showed that CDK5 played an important role in cancer tumorigenesis and progression. For example, CDK5 has been reported to highly express in hepatocellular carcinomas and promote tumor vessels formation though directly stabilizing the HIF-1α [9, 10], and CDK5 also participated in regulating the migration of prostate cancer cells [11]. Furthermore, the association between high CDK5 expression and poor prognosis has showed in other human malignancies, such as pancreatic cancer [12], lung cancer [13], thyroid carcinoma [14]. While, Sun Y et al. reported that lower expression of CDK5 associated with poorer prognosis in gastric cancer [15]. Yet, there is no research about CDK5’s function which focusing on ccRCC patients. As is known to all, pVHL targets two main a-subunits of hypoxia-inducible transcription factors (HIFs), including HIF-1α and HIF-2α, unlike many vascularized solid tumors, HIF-1α has opposing effects in ccRCC compared with HIF-2α. HIF-1α acts as a tumor suppressor, while HIF-2α acts as an oncogene on ccRCC development and progression [16].

CDK5 has been demonstrated to stabilize HIF-1α in hepatocellular carcinoma, which is also one of the most vascularized tumors. We hypothesize whether CDK5 has the same effects in ccRCC, but acting as tumor suppressor. Recent studies have revealed that CDK5 suppressed the activities of several cell cycle inhibitors such as p21 and p27, and leading to the over proliferation of cancer cells [17]. p21 encoded by CDKN1A is a well-known tumor suppressor that participate in regulating cell proliferation [18]. Our previous study has found that inhibition of the LSD1 decreased the H3K4 demethylation at *CDKN1A* gene promoter, which was associated with the p21 upregulation and cell cycle arrest at G1/S in ccRCC cells [19]. And the RNA-sequence result showed CDK5 upregulating as well as p21 (Additional file 1: Figure S1), so we also hypothesize that CDK5 and p21 may have functional correlation in predicting ccRCC patients’ prognosis.

## Methods

### Patients and samples

This study enrolled 150 patients with ccRCC, who underwent nephrectomy at Zhongshan hospital, Fudan University from 2008 to 2010, including 107 male and 43 female (mean age 57.0 years). Clinical and pathological information (e.g. age at surgery, gender, tumor size, pathology, TNM stage, Fuhrman degree, and all necessary follow-up messages.) for all participants included in this study were collected and then evaluated. 10 paired fresh and frozen ccRCC samples were randomized collected in my department for quantitative real-time PCR and Western bolt analysis, and patients’ information were showed in Additional file 1: Table S1. The study was performed with the approval of the ethics committee of Zhongshan hospital. Written informed consent for each participant was obtained.

### Immunohistochemistry

Tissue microarrays (TMAs) were made using above 150 tissues in Shanghai Outdo Biotech Company (Shanghai, China) including tumor tissue and adjacent tissues. The immunohistochemistry (IHC) was performed by the streptavidin-peroxidase method (Zymed Laboratories Inc., San Francisco, CA, USA). The CDK5 antibody was purchased from abcam (ab40773, Cambridge, MA, USA) and diluted into 1:50. The p21 (CDKN1A) antibody was purchased from Cell signaling technology (CST) (mAb#2947, Danvers, MA, USA) and diluted into 1:50 as well. CDK5 and p21 IHC score were determined by both the intensity and percentage of tumor cell. The intensity of staining was classified as 0 (negative), 1 (weak), 2 (moderate), 3 (strong), and the percentage was assigned as following: 1 (0–25%), 2 (26–50%), 3 (51–75%), 4 (> 75%) (Additional file 1: Figure S2). The total IHC staining score was calculated by intensity × percentage. The IHC score below six was defined as low expression group, while score over six was defined as high expression group. Immunostaining was assessed and examined independently by two observers (LS. Zhu and R. Ding).

### RNA extraction and quantitative RT-PCR

The total RNA was extracted from 10 paired fresh and frozen ccRCC samples (100 mg each) with Trizol (1 ml), and add chloroform (200ul) according to the standard protocol with RNase free condition. And the quantification of mRNAs was performed with the SYBR Green kit (Takara Bio, Dalian, China). As following: 2X SYBR 10ul; Fp/Rp 0.5/0.5 ul; Template 1ul (or ddH_2_O 1ul as negative control); ddH_2_O 8ul.The PCR protocol was following: Predenature 95 °C 2 min; Denature 95 °C 10 s, Annealing 57 °C 30 s and Extension 72 °C 45 s for 35 cycles (BIO-RAD CFX Connect™ Real-Time System, Hercules, USA). And the qPCR data was analyzed using the 2^-ΔΔCt^ method normalized to GAPDH. CDK5 expression was presented as the fold change. PCR primers were listed as follows: human CDK5, forward 5′-AATGACTGGGAGGAGAGAGGGAG-3′, reverse 5′- TTCACGGCGTGCATACTCAG-3′; human GAPDH, forward 5′-ACAGTCAGCCGCATCTTCTT-3′ and reverse 5′-GACAAGCTTCCC GTTCTCAG-3′.

### Western blot assay

Western bolt procedure was performed with the protein lysates obtained form fresh tumor samples according to our previous research [19]. Equal amounts of protein samples were resolved in 10% SDS-PAGE (Bio-Red Laboratories, Inc.). Then, protein was transferred to polyvinylidene difluoride (PVDF) membranes (Bio-Red Laboratories, Inc.). After blocking with 3% Bovine serum Albumin (BSA) for 1 h at room temperature, the membranes were separately incubated with primary anti-CDK5 antibody (1:1000; abcam ab40773, Cambridge, MA, USA.) and anti-GAPDH antibody (1:1000; CST mAb#5174, Danvers, MA, USA.) overnight at 4 °C. Following, the membranes conjugated secondary antibody for 1 h. The signal intensity was evaluated using an enhanced chemiluminescence system (GE Healthcare Life Science, Chalfont, UK.).

### Database analysis and survival data

The CDK5 expression level in kidney cancer was examined from both TCGA (The Cancer Genome Atlas) (https://cancergenome.nih.gov) and GENT (Gene Expression of Normal and Tumor tissues) databases (http://medicalgenome.kribb.re.kr/GENT/). The Oncomine database (http://www.oncomine.org) is a web-based database platform that incorporates 264 independent datasets and aims to collect, standardize, analyze, and deliver transcriptomic cancer data for biomedical research. We searched the key words, Gene: CDK5/p21; Analysis type: Cancer vs Normal Analysis; Cancer type: Kidney Cancer. And we set the detailed dataset, grouped by overall survival status (days). The overall survival rate was measured according to this data of 452 patients as a validation cohort.

### Statistical analysis

Statistical analysis was performed using IBM SPSS statistical version 19.0. The pathological and clinical characteristics of the two groups in all cases were assessed by the χ2 test or Fisher’s exact test. Survival analysis was performed using the Kaplan-Meier method and compared with a log-rank test. The Cox proportional hazards regression model was used for determining the significant prognostic factors. All *p* values were 2-sides and those less than 0.05 were defined significant.

## Results

### Expression of CDK5 and p21 in ccRCC patients

**Fig. 1:**
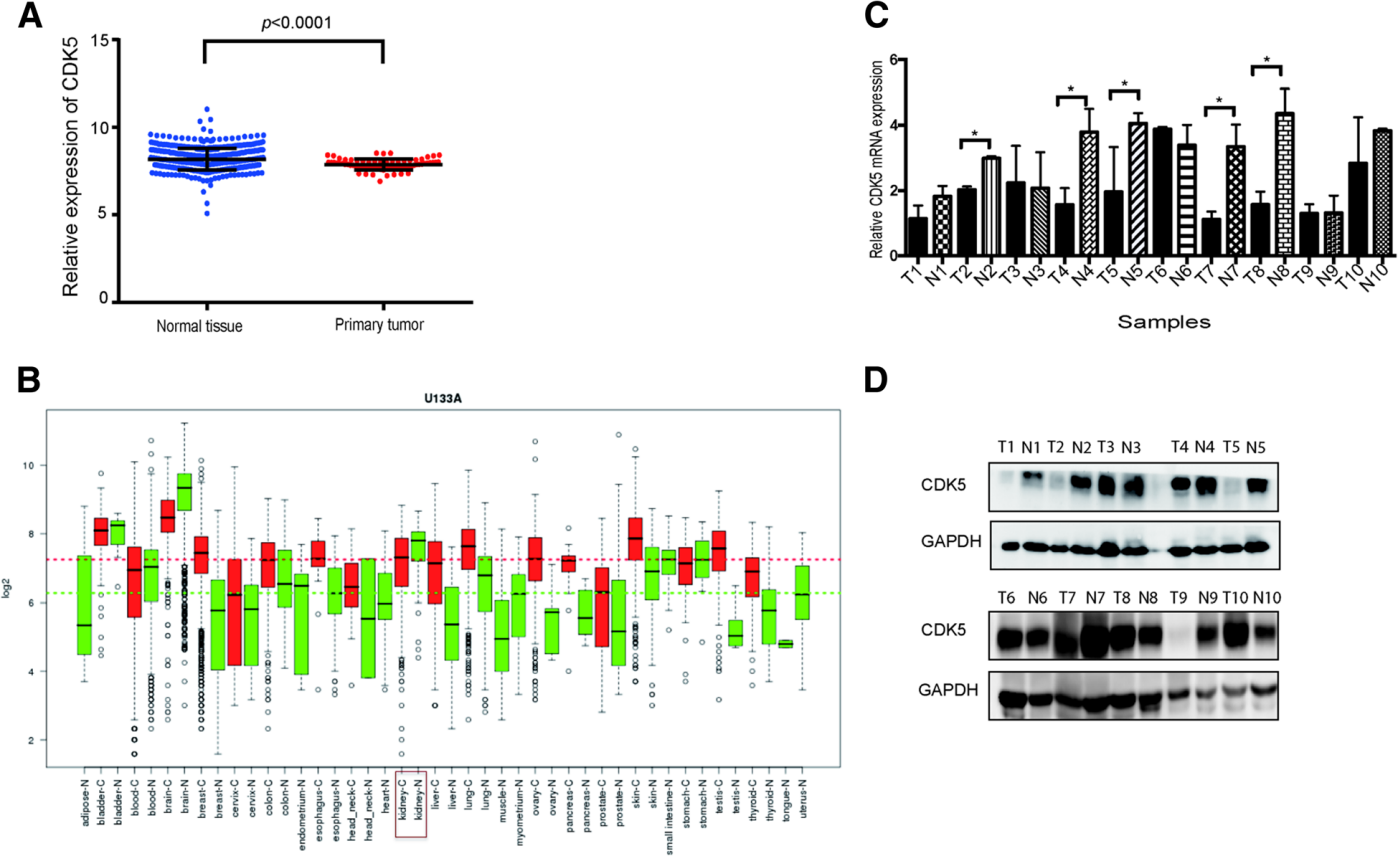
The expression of CDK5 in kidney cancer. **a** The CDK5 expression among normal tissue and primary renal tumor in TCGA database, showed lower CDK5 expression in cancer tissues (*p* < 0.0001). **b** The CDK5 expression among kidney cancer and normal tissue in GENT database. **c** Ten paired fresh ccRCC samples were tested the CDK5 mRNA expression by using qRT-pcr (T means tumor tissue and N means matched normal tissue). **d** The protein levels of CDK5 were tested by western blot in same ccRCC samples. *p < 0.05

**Fig. 2:**
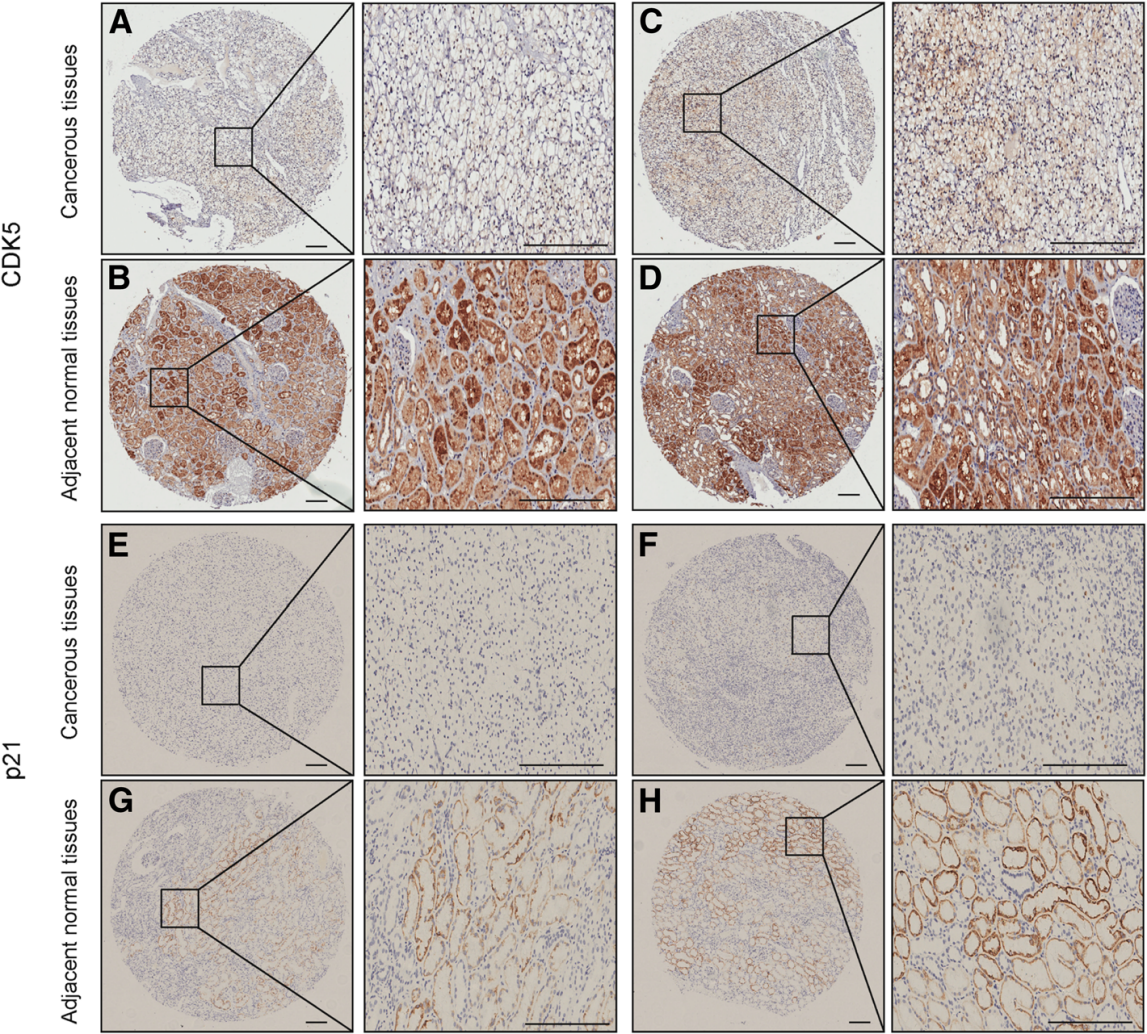
Representative immunohistochemical staining images of CDK5 and p21 in 2 patients’ ccRCC and adjacent normal tissues. **a**-**d** The strong staining of CDK5 in normal tissue and lower staining in cancerous tissues. **e**, **g**. **f**, **h**. The strong staining of p21 in normal tissue and lower staining in cancerous tissues. Bar,100 um

Firstly we compared the CDK5 mRNA expression in kidney cancer from both TCGA and GENT databases (Fig. 1a, b). We found that the CDK5 expression was significant lower in primary cancer tissues compared with adjacent normal tissues in TCGA (*p* < 0.0001). And the GENT database also showed the similar result. This result was recapitulated in fresh ccRCC samples by using qPCR assay as well (Fig. 1c). The western blot assay also showed that the protein level of CDK5 were relatively higher in normal tissues compared with the cancer tissues (Fig. 1d). Then we examined the CDK5 and p21 protein expression in ccRCC TMAs using IHC. As shown in Fig. 2, CDK5 staining showed in the cytoplasm and nuclei, while p21 localized only in the nuclei of ccRCC. CDK5 was highly detected in adjacent normal tissues than paired cancerous tissues as well as p21, with different staining intensity in different specimens. The results showed that CDK5 and p21 expression were downregulated in ccRCC compared with normal tissue.

The CDK5 expression was found to be as low group in 83 patients (55.3%), and high in 67 patients (44.7%). The p21 expression was scored as low in 97 patients (64.7%), and high in 53 (35.3%). We also classified the patients into some types according to the combination expression of CDK5 and p21 as following: CDK5 low and p21 low group (CLPL *n* = 56), CDK5 high and p21 high group (CHPH *n* = 26), CDK5 low and p21 high group (CLPH *n* = 27), and CDK5 high and p21 low group (CHPL *n* = 41).

### Relationship between clinicopathological characteristic and CDK5/p21 expression in ccRCC patients

The patients’ clinicopathological characteristics were analyzed (Table 1). 107 (71.3%) male and 43 female (28.7%) were included in this study. Eighty four patients (56%) were older than 55 years. Among all cases, the distributions of TNM stage I + II and III + IV, accounting for 138 (92%), and 12 (8%) respectively. As the Fuhrman grade, 108 patients (72%) were classified as grade I + II, while 42 patients were classified as grade III + IV. The CDK5 expression was significantly associated with advanced TNM stage (*p* = 0.042), and Fuhrman grade (*p* = 0.035). Patients with lower expression of CDK5 may more likely have worse outcome. The expression of p21 was significantly related to Fuhrman grade (*p* = 0.026) as will. Patients in the group of lower p21 expression showed high rate of Fuhrman grade III + IV. And no significant difference was observed in other factors.

**Table 1:** Baseline characteristic

Characteristic		Patients		Tumoral CDK5 expression			Tumoral p21 expression		
		n	%	Low	High	*P*-value	Low	High	*P*-value
All patients		150	100	83	67		97	53	
Gender						0.661			0.289
	Male	107	71.3	58	49		72	35	
	Female	43	28.7	25	18		25	18	
Age (years)						0.624			0.356
	≤55	66	44	38	28		40	26	
	> 55	84	56	45	39		57	27	
TNM stage						0.042			0.172
	I + II	138	92	73	65		88	50	
	III + IV	12	8	10	2		10	2	
pT stage						0.066			0.435
	T1 + T2	139	92.7	74	65		89	50	
	T3 + T4	11	7.3	9	2		8	3	
pN stage						0.439			0.279
	N0	147	98	82	65		96	50	
	N1	3	2	1	2		1	2	
pM stage						___			___
	M0	150	100	84	66		97	53	
	M1	0	0	0	0		0	0	
Fuhrman grade						0.035			0.026
	I + II	108	72	54	54		64	44	
	III + IV	42	28	29	13		33	9	
Tumor size (cm)						0.250			0.393
	≤4	75	50	45	30		51	24	
	> 4	75	50	38	37		46	29	

### Lower CDK5 and p21 expression proved to be independent prognosis factor in ccRCC

**Fig. 3:**
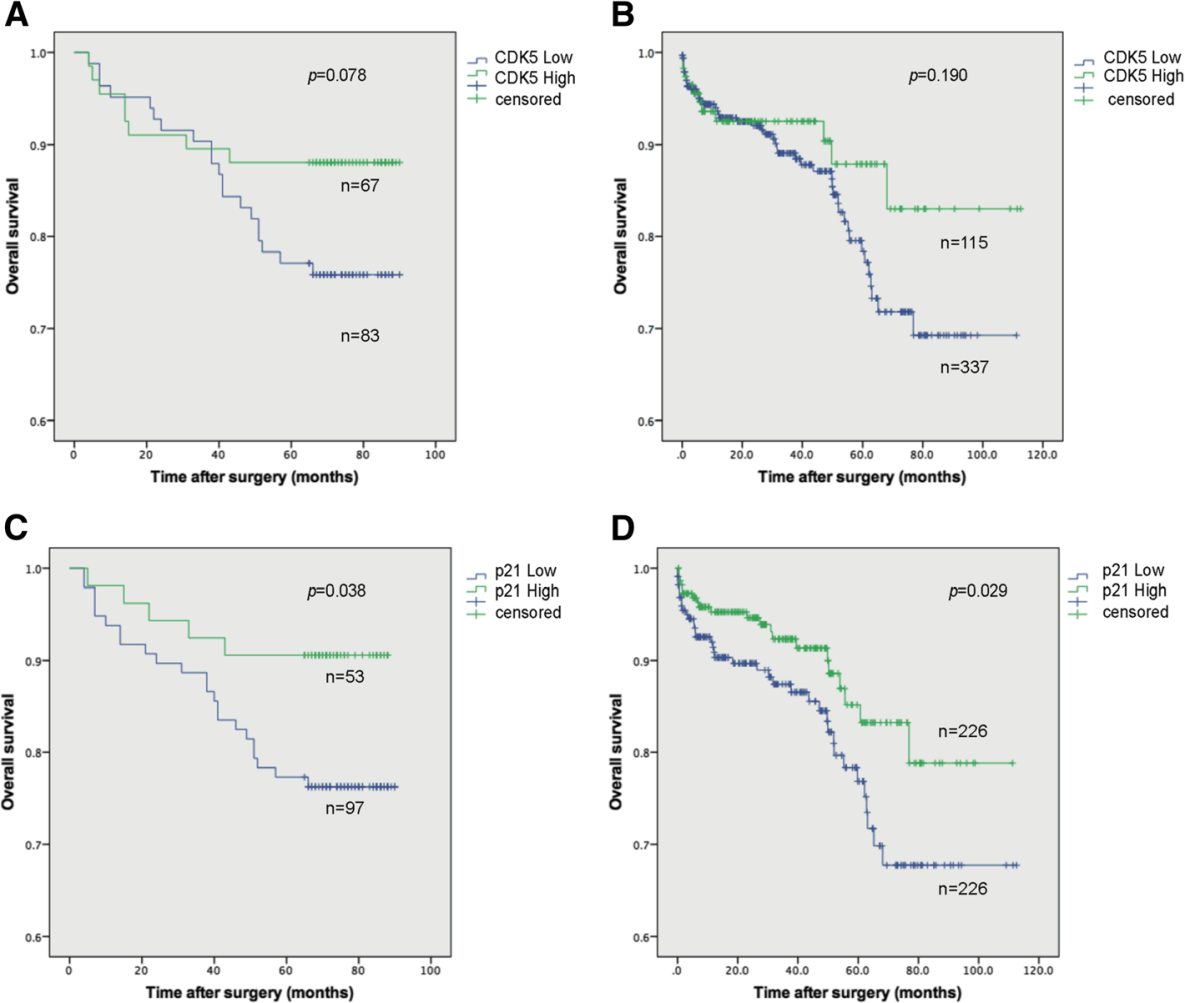
The overall survival curves based on the CDK5 and p21 expression in ccRCC patients. **a**-**c** showed the survival results based on the TMAs, and **b**-**d** showed the survival results based on the Oncomine database

**Fig. 4:**
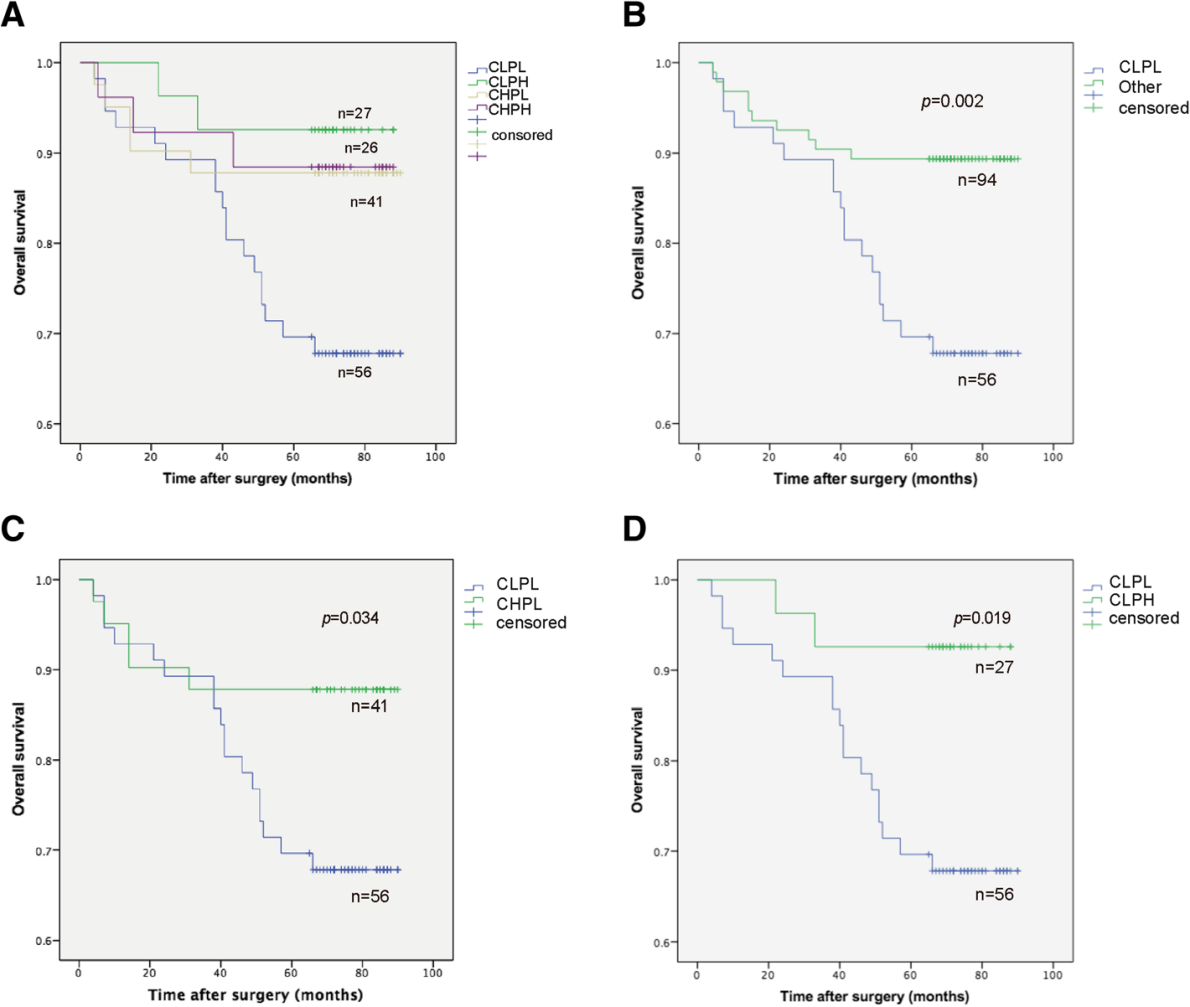
The overall survival curves based on the combined CDK5 and p21 expression in ccRCC patients. **a** Different group of Patients in OS analysis. **b** CLPL group was compared with the other groups. **c** The OS analysis of patients in CLPL group and CHPL group. **d** The OS analysis of patients in CLPL group and CLPH group

In order to evaluate prognostic value of the expression of CDK5 and p21, Kaplan-Meier survival curves and log-rank tests were performed. As presented in Fig. 3a, the 3-year and 5-year survival rates in CDK5 low group were 83.1 and 69.9%, and the rates became 89.6 and 88.1% in CDK5 high group. The validation cohort showed that the 3-year and 5-year survival rates in CDK5 low group were 91.1 and 87.5%, and 93.0 and 91.3% for those with high CDK5 expression (Fig. 3b). Though, no significant difference was observed in these two cohorts, patients with CDK5 low expression were more likely have worse survival. The same 3-year and 5-year survival rates in p21 low expression patients were 86.7 and 79.4%, 92.5 and 90.6% for those with high p21 expression (*p* = 0.038) (Fig. 3c). The validation cohort also showed the significant difference in OS rate in p21 low and high expression (*p* = 0.029) (Fig. 3D). We further evaluated the combined prognostic value of CDK5 and p21 expression. As presented in Fig. 4, CLPL group patients have worse OS rate (*p* = 0.002), and no significant difference was observed in other group.

Further, the univariate and multivariate analyses were used to determine the independent prognostic factors for ccRCC patients (Table 2). In univariate analysis, the p21 expression level instead of CDK5 was found to be significantly associated with the OS (*p* = 0.047). And the CLPL group showed significant association as well (*p* = 0.003). Other factors such as age (*p* = 0.010), TNM stage (*p* < 0.001), Fuhrman grade (*p* = 0.001), and tumor size (*p* = 0.002) were also correlated significantly with OS. Moreover, multivariate analysis showed that CLPL (*p* < 0.001), TNM stage (*p* = 0.027), tumor size (*p* = 0.011) were proven to be independent predictors of OS for ccRCC patients.

**Table 2:** Summary of univariate and multivariate Cox regression analysis of OS duration in all ccRCCs

Variables	Univariate analysis			Multivariate analysis		
	HR	(95% CI)	p*	HR	(95% CI)	p*
CDK5 expression (low vs high)	0.486	0.214–1.104	0.085			
p21 expression (Low vs High)	0.375	0.142–0.985	0.047	0.369	0.162–0.839	0.078
CDK5/p21expression (CLPL vs Other)	0.310	0.143–0.672	0.003	0.229	0.102–0.515	< 0.001
Age (< 55 vs > 55)	1.047	1.011–1.084	0.010	1.022	0.986–1.060	0.237
TNM stage (I + II vs III + IV)	6.972	2.935–16.563	< 0.001	2.920	1.129–7.556	0.027
Fuhrman grade (I + II vs III + IV)	5.043	2.359–10.781	0.001	3.299	0.776–6.254	0.138
Tumor size (< 4 vs > 4)	4.247	1.721–10.479	0.002	3.490	1.329–9.164	0.011

CLPL CDK5 low/p21 low, HR hazard ratio, 95% CI 95% confidence interval

## Discussion

Currently, RCC patients who have underwent the nephrectomy, usually need to take regular follow-up examinations, including blood test, computed tomography annually, in order to find recurrences as soon as possible and predict the long-term outcomes. The biomarker for evaluating and predicting the prognostic survival is still limited, so it is necessary to find new effective biomarker. In this study, we firstly demonstrated the notable association between low CDK5 expression and advanced ccRCC pathological features. Though, there was no significance observed in OS outcome, patients with low CDK5 expression seem to have poor prognosis. And the co-expression of CDK5 and p21 were also proven to be an independent prognostic factor in ccRCC patients.

The functional roles of CDK5 were well studied in the nervous system because it was discovered and characterized initially in the brain tissues [20]. CDK5 performs its significant role not only in the natural development of nervous system but also as a passive promoter during the development of pathological neurology disease [21]. Recently, a growing number of articles were focusing on the role of CDK5 in extra-neuronal oncology. Increasing researches have revealed that the abnormal expression of CDK5 participated in the tumor progression and metastasis across various solid malignancies, including breast cancer, lung cancer, prostate cancer, and liver cancer [22]. To date, the research of CDK5 in kidney cancer still limited, and our study focused on the expression of CDK5 in ccRCC patients, which is the most common pathological type.

p21 expression and protein activities are modified by multiple mechanism. It has been demonstrated that p21 acts as a well-known tumor suppressor in various types of cancers, because p21 is one of the most important target in p53 signaling pathway and functioned as cell-cycle checking point to inhibit cancer cell over proliferation [23]. In our previous work, we also found that inhibition of LSD1 would suppress the ccRCC growth through upregulating p21 signaling. Lately, Pao-Hsuan Huang and colleagues reported CDK5 could directly target p21, and overexpression of CDK5 triggered the degradation of p21 and promoted several cancer cells (breast cancer, prostate cancer, lung cancer) growth [24]. However, no studies have focused on the prognostic values of CDK5 and the combination of CDK5 and p21 in ccRCC patients. In this study, we found that both CDK5 and p21 were downregulated in cancer tissues compared with normal side, and lower CDK5 expression was significantly associated with advanced TNM stage (*p* = 0.042), and Fuhrman grade (*p* = 0.035), lower p21 was significant associated with Fuhrman grade (*p* = 0.026) either. What’s more important, the patients with combination of low CDK5 and low p21 (CLPL) showed poorer survival than other groups in Fig. 4b. CLPL patients also have worse survival compared with CHPL group (*p* = 0.034) and CLPH group (*p* = 0.019) respectively. No significant OS rate difference was observed in other groups. It’s worth mentioning that the patients in CDK5 high and p21 high group seem to have a better outcome (Fig. 4b), but the patients’ number is so limited that larger sample is needed.

Also, we suggested that the combination value of CDK5/p21 might integrate to the current model in predicting survival of ccRCC patients as an independent prognostic factor. However, large sample of patients is still needed to verify the prognostic value of CDK5/p21, and the basic mechanism within CDK5 and p21 is required in future studies.

## Conclusion

Taken together, in our study, we tested the expression of CDK5 and the combination of CDK5 and p21 in ccRCC samples in the first time. The data suggested that the both CDK5 and p21 were acting as promising biomarkers in ccRCC patients, and CDK5/p21 is closely associated with worse pathological outcome. This may provide the new target for therapeutic intervention in ccRCC patients.

## Additional file

Additional file 1: **Figure S1.** The mRNA expression of specific gene after LSD1 inhibition in ccRCC cell lines (RNA-seq data). **Figure S2.** Representative images of IHC staining of CDK5 and p21. Bar 100um. **Figure S3.** The comparation of overall survival rate of CHPH patients with others. **Table S1.** Patients’ information of the fresh samples. Age (range 42–72 years old). (DOCX 9 kb)

## Data Availability

The datasets used and analyzed during the current study are available from the corresponding author on reasonable request.

## Abbreviations

ccRCC: clear cell Renal Cell Carcinoma
CDK5: cyclin-dependent kinase 5
CHPH: CDK5 high and p21 high group
CHPL: CDK5 high and p21 low group
CLPH: CDK5 low and p21 high group
CLPL: CDK5 low and p21 low group
GENT: Gene Expression of Normal and Tumor tissue
HIF: Hypoxia-Inducible transcription Factor
IHC: Immunohistochemistry
OS: Overall Survival
RCC: Renal cell carcinoma
TCGA: The Cancer Genome Atlas
TKIs: Tyrosine Kinase Inhibitors
TMA: Tissue Microarray
VHL: Von Hippel-Lindau
