# Supplementary material for: Cyclin-dependent kinase 5 acts as a promising biomarker in clear cell Renal Cell Carcinoma
Source: BMC Cancer. 2019 Jul 16;19:698. doi: 10.1186/s12885-019-5905-9 (PMC6636025; doi:10.1186/s12885-019-5905-9)
Supplement: Supplementary file 1 — Figure S1. The mRNA expression of specific gene after LSD1 inhibition in ccRCC cell lines (RNA-seq data). Figure S2. Representative images of IHC staining of CDK5 and p21. Bar 100um. Figure S3. The comparation of overall survival rate of CHPH patients with others. Table S1. Patients’ information of the fresh samples. Age (range 42–72 years old). (DOCX 9 kb) [file 12885_2019_5905_MOESM1_ESM.docx]

**Supplementary**

**Figure S1**

The mRNA expression of specific gene after LSD1 inhibition in ccRCC cell lines (RNA-seq data).

**Figure S2**

Representative images of IHC staining of CDK5 and p21. Bar 100um.


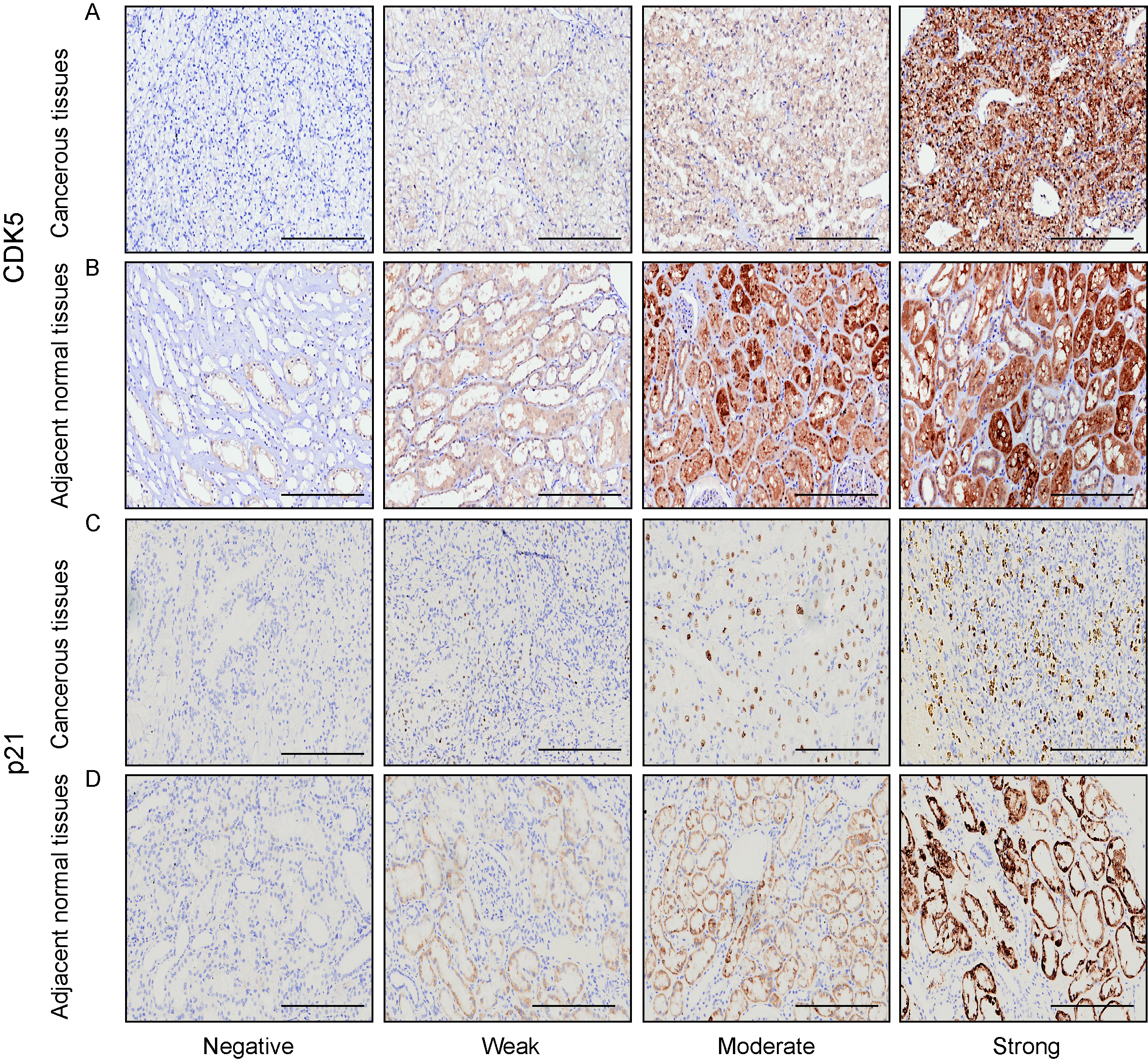


**Figure S3**

The comparation of overall survival rate of CHPH patients with others.


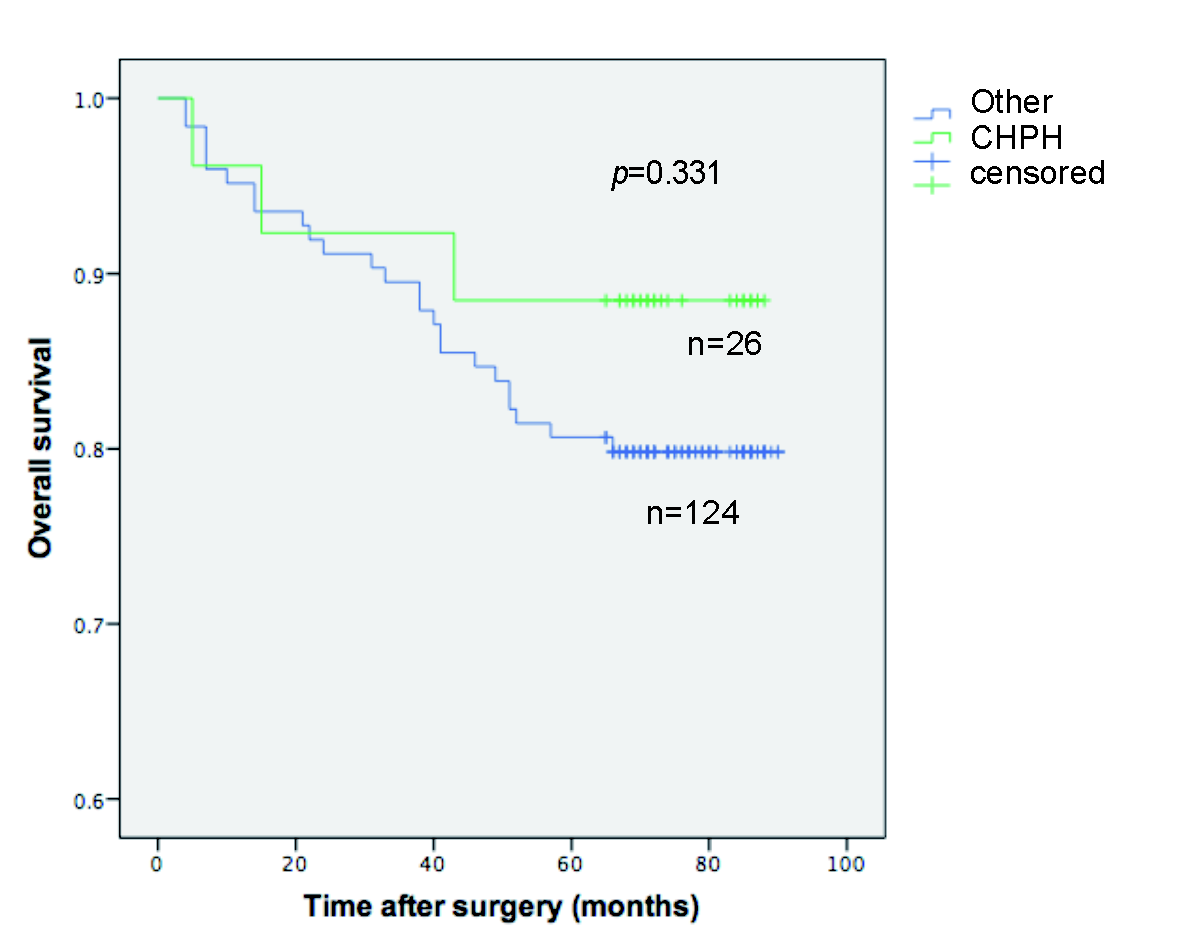


**Table S1**

Patients' information of the fresh samples.

| Patient | CDK5 expression | Fuhrman grade | Tumor size (cm) | TNM stage |
| --- | --- | --- | --- | --- |
| 1 | Low | II | 4.8 | II |
| 2 | Low | III | 5.7 | III |
| 3 | high | II | 4.2 | II |
| 4 | Low | II | 4.0 | I |
| 5 | Low | II | 5.8 | III |
| 6 | High | I | 3.6 | I |
| 7 | Low | III | 4.7 | II |
| 8 | high | II | 4.2 | II |
| 9 | Low | III | 6.9 | III |
| 10 | high | I | 3.6 | I |

Age (range 42-72 years old)
